# Accurate Magnetic Sensor System Integrated Design

**DOI:** 10.3390/s20102929

**Published:** 2020-05-21

**Authors:** Nicolò Marconato, Roberto Cavazzana, Paolo Bettini, Andrea Rigoni

**Affiliations:** 1 Consorzio RFX, Corso Stati Uniti 4, 35127 Padova, Italy; roberto.cavazzana@igi.cnr.it (R.C.); paolo.bettini@unipd.it (P.B.); andrea.rigoni@igi.cnr.it (A.R.); 2 Department of Industrial Engineering, University of Padova, 35131 Padova, Italy

**Keywords:** magnetic measurements, magnetic sensor, inductive probe, integrator drift, noise, plasma, nuclear fusion

## Abstract

Inductive measurement of magnetic fields is a diagnostic technique widely used in several scientific fields, such as magnetically confined fusion, plasma thrusters and particle accelerators, where real time control and detailed characterization of physics phenomena are required. The accuracy of the measured data strongly influences the machine controllability and the scientific results. In the framework of the assembly modifications of the RFX-mod experiment, a complete renew and improvement of the magnetic diagnostic system, from the probes moved inside the vacuum vessel to the integrator modules, has been carried out. In this paper, the whole system making up the magnetic diagnostics is described, following the acquisition chain from the probe to the streamed data and illustrating the requirements and conflicting limitations which affect the different components, in order to provide a comprehensive overview useful for an integrated design of any new systems. The characterization of a prototypical implementation of the whole acquisition chain is presented, focusing on the flexible ADC architecture adopted for providing a purely numerical signal integration, highlighting the advantages that this technology offers in terms of flexibility, compactness and cost effectiveness, along with the limitations found in existing implementation in terms of ADC noise characteristics and their possible solutions.

## 1. Introduction

Magnetic field measurements are extensively used in the domain of magnetically confined fusion [1,2,3], plasma thruster [4] and particle accelerator experiments [5]. These measurements are needed both in real time, to feed-back magnetic field information for real time control of various machine systems, and for data analysis required in physics study [6,7,8]. A measurement system for these applications has very demanding requirements: high resolution in both time and space, hundreds of kHz of bandwidth, wide dynamic range, increasingly long time acquisition and relative accuracy up to 100 ppm. A single measurement system can hardly satisfy all such challenging needs at the same time. In particular, the choice of inductive sensors is forced by the typical magnitude and frequency bandwidth of the magnetic field characterizing the phenomena of interest, along with the harsh environment where the measurement probes are often installed.

Many papers address inductive sensors and their applications [9]. In this paper, we systematically outline the particular features, requirements and hurdles in designing an integrated system of distributed sensors, able to precisely characterize fast magnetic transient phenomena in both space and time. The paper describes the whole measurement chain, underlining the different issues encountered along the entire process from the inductive sensor to the streamed data, and provides the description of a compact and cost effective digital integration system designed for the new electromagnetic measurement for the second upgrade of the Reverse Field eXperiment (RFX-mod2). Indeed, in the new experiment configuration, all electromagnetic sensors are moved inside the stainless steel vacuum vessel, widening their usable signal bandwidth up to 200 kHz and assuring better plasma control. Moreover, the total number of the new magnetic pickup coil sensors are increased and upgraded to improve spatial resolution [10]. By taking this new system as a reference, the different requirements, parameters affecting the performance and conflicting limitations are highlighted along with the complexity in managing a high number of sensors and channels. Moreover, a thorough description of the issues related to voltage integrator drift on long lasting signals and the need to customize the system in a real time framework are presented, with the aim of providing a complete and compact set of guidelines for the integrated design of any inductive magnetic measurement system for other similar applications.

The paper is organized as follows. In Section 2, the elements constituting the acquisition chain are described, focusing on the requirements of the final measurement system and the issues to achieve them due to the limitations introduced by different components. Section 3 introduces the need of the signal integration, highlighting the pros and cons of analog and digital methods, in particular related to the detrimental drift problem adversely affecting the system even for short-term applications. Section 4 presents the characterization of a prototypical implementation of the whole acquisition chain adopted for our application. Finally, Section 5 presents the flexible Analog to Digital Converter (ADC) architecture adopted. In particular, this solution provides support for the purely numerical signal integration, sub-sampling for real-time streaming, region of interest (ROI) detection and timing extraction. In our description, we focus on one side on the opportunity that this technology offers in terms of flexibility, compactness and cost effectiveness, and, on the other side, we discuss the limitations found in the existing implementation in terms of ADC noise characteristics, along with possible solutions. Even if applied to this specific case, the results obtained here could be used as guidelines for other similar applications as well.

## 2. Magnetic Measurements

Inductive sensors are a widely used means of measuring magnetic fields and related quantities, having the important advantages to be linear up to the range of hundreds of kHz and are easily calibrated accurately. These consist of different forms of conducting loops, each measuring the voltage induced by a changing magnetic field. Inductive sensors are simple and inexpensive, allowing many channels to be used for spatially detailed measurements. Despite the wide variety of applications, all of these measurements are based on the principle of a simple inductive loop. Essentially, three different kind of inductive sensors can be distinguished. Generally, the word “loop” denotes a large size probe made of a small number of turns for magnetic flux or averaged field measurement. Pick-up or Mirnov coils are small size coils with a large number of turns for localized magnetic field measurements. The so-called Rogowski coils are part of the inductive sensor category as well, and they consist of a particular winding around a closed path allowing the measurement of currents flowing inside that path.

According to Faraday’s law, the voltage difference *V* at the terminals of a *n* turn conducting loop is equal to the rate of change of the magnetic flux Φ that is linked by the single coil turn:

(1) $$V=-n\frac{d\Phi }{dt}.$$

Therefore, the integration of the voltage signal *V* from the coil with the known initial conditions ($\Phi_{0}$) gives a direct measurement of the magnetic flux that is under observation: $\Phi =\Phi_{0}-\int Vdt$.

Therefore, the key parameter for each of the above mentioned types of inductive sensor is the relationship between the flux Φ linked by the loop and the magnetic quantity of interest. Hereinafter, only pick-up coils will be taken into account and mentioned also as magnetic probes or magnetic sensors.

Considering Equation (1), it is clear that a voltage signal in the sensor output is associated to a time variation of the flux density. However, by using a large area sensor and sensitive amplifier, very low frequency magnetic fields (of several mHz) can be detected.

It is well known that the larger the equivalent area (large active area and/or large number of turns) the higher the coil sensitivity. However, the optimization of the coil performance certainly is not a trivial process, characterized by a trade-off among several conflicting requirements, such as sensitivity, frequency spectrum, spatial and time resolution, and limiting constraints, such as available room, distances to be covered by transmission lines, etc.

**Figure 1:**
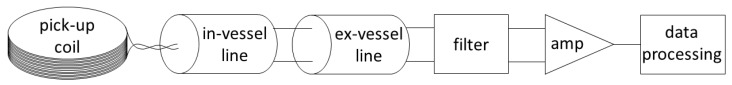
Schematic diagram showing the pick-up coil connected via in-vessel and ex-vessel transmission line sections to a data processing system.

For example, magnetic sensors intended for both plasma active control and magneto- hydrodynamics (MHD) study need to be installed inside the vacuum chamber in order to allow measurements of actual magnetic field fluctuations. This will necessarily impose limitation on the coil size, as well as limitation on the allowed materials. A single system capable of meeting both the purposes needs a high time resolution and a broad frequency range, from 0 to 100s kHz (very high-frequency phenomena necessarily require ad hoc probes). As examined in depth in Section 2.1, this is in conflict with the required high sensitivity which would lead toward a large number of turns or a large probe area. A wide coil in turn conflicts with the spatial resolution requirement and in addition usually its dimensions are limited by the available space. In a practical application, the connection from the terminals of each loop to the electronic instrumentation is made using a twisted pair or coaxial cables in order to minimize the cables contribution to the total magnetic flux intercepted by the circuit and thus maximize the signal to noise ratio due to environmental causes. Because of the typical harsh operating conditions within the vacuum vessel, different cables are usually used in the cable path sections inside and outside the vessel and thus their electrical properties are not necessarily the same. Generally, at high frequencies, the inhomogeneity of the cables might become important due to transmission line effects. In Figure 1, a schematic representation of the whole magnetic measurement system is shown. The effect of the connecting cable may be neglected or at most be included as an effective capacitance when the detection of frequencies below 50 kHz is of interest [11]. Above this frequency range, the contribution of the transmission line has to be carefully considered.

Magnetic probe signals might require to be pre-amplified near the machine before the long transmission line. Then, they are either amplified to be used as the time derivative of the field or integrated to obtain the field itself. Some channels can also require to be attenuated in order to study fast events which would lead to saturation of amplifiers or ADCs. Signals are generally passed through a low pass passive filter along the acquisition chain as well. Each of these elements, represented in Figure 1, are described in the following subsections.

### 2.1. Inductive Sensor Model

Magnetic pickup coils are used to detect magnetic phenomena with frequencies ranging from a few Hz to many MHz. However, size and shape affect the frequency response of the probe. While the effective area $A_{eff}$ increases linearly with the number of turns *N*, its inductance $L_{p}$ increases quadratically; therefore, as treated in detail in Section 2.1.1, the bandwidth of the measurement decreases inversely with the probe sensitivity. In particular, the self-inductance of an air-core coil can be expressed by the general form

(2) $$L=\frac{\mu_{0}N^{2}A_{c}}{l}K.$$

where $A_{c}$ is the coil cross section, *l* is its axial length and K(d,a,b,l) is a shape factor, again a function of the coil length *l*, cross dimensions *a* and *b* and wire diameter *d* [12]. At constant $A_{c}$, *d* and *l*, taking as a reference the inductance of a circular coil $L_{circ}$, the shape factor for a square coil is about $K_{square}=1.2\cdot K_{circ}$ and it increases with the aspect ratio ρ for a rectangular coil. For the circular coil of radius *r*, the following approximate formula is used:

(3) $$L_{circ}=N^{2}\mu_{0}\frac{D}{2}\cdot \left(\ln\frac{8a}{d}-7/4\right).$$

The rectangular coil formula instead is:

(4) $$L_{rect}=\frac{\mu_{0}}{\pi }\left[-2\left(a+b\right)+2\sqrt{a^{2}+b^{2}}-a\ln\frac{a+\sqrt{a^{2}+b^{2}}}{b}-b\ln\frac{b+\sqrt{a^{2}+b^{2}}}{a}+a\ln\frac{2a}{d/2}+b\ln\frac{2b}{d/2}\right].$$

A possible way to improve sensitivity without limiting the bandwidth is to increase the actual cross section of the coil $A_{c}$. As treated in some detail in Section 2.2, minimizing the capacitance *C* of the cable connecting the coil to the termination will also help to maximize the bandwidth.

The dynamic range requirements for the signal acquisition system demands a careful evaluation of both the minimum detectable and the maximum expected signal, in particular when its numerical integration is considered. The maximum level of the expected signals has to be somehow estimated and will define in particular the filter and attenuator design. On the other hand, the minimum signal requirement involves the sensitivity to slowly changing magnetic fields at low intensity and thus constrains in particular the coil effective area and the noise rejection of the system and possible amplifiers. The minimum required signal amplitude implies an effective probe area typically of a few hundred square centimeters, which can also allow bandwidth on the order of some hundreds of kHz. High bandwidth measurements of fast phenomena are obtained by high frequency pick up coils usually based on a special design that guarantees high bandwidth, typically higher than 300 kHz.

In addition, the magnetic probe size and shape are in general a compromise between the required effective area of the winding and the available space. Indeed, for several reasons, the sensors should be as close as possible to the magnetic source which often means in a vacuum, narrow and harsh environment. Highly compact, radiation hard magnetic probes have been built, for example, by employing a thick film technology [13], to build a sandwich of planar coils of conducting material printed in insulating ceramic sheets stacked one on the top of an other and connected by auxiliary circuits.

It is worth noting that the electrical parameters of the probe are temperature dependent. Indeed, the coil inductance tends to increase with increased temperature, as well as the cable resistance, because of the thermal expansion of the copper which the coil cable is made of. The design of probes for high temperature operation (even several hundred degrees Celsius) must take particular care in the selected material, especially in terms of relative thermal expansion, which may significantly change the effective area of the probe. In particular, the material adopted for the former which the winding is wrapped around has to show a similar thermal expansion to that of the adopted cable, since repeated relative expansions lead to a change in the electrical characteristics of the coil and the consequent loss of calibration [14].

A further ploy in magnetic probe design is the choice of an even number of layers in order to minimize the collecting area in perpendicular directions with respect to the coil axis and therefore the cross talk with perpendicular magnetic fields.

#### 2.1.1. Sensor Sensitivity and Frequency Response

The ideal circuit model of an air-core inductive sensor consists of a linear inductor with self-inductance $L_{p}$ subject to an electromotive force $V_{in}$ linked to the measured magnetic flux according to Equation (1). By assuming the magnetic flux varies as a sine wave, Equation (1) implies that for the ideal sensor the output signal, or in other words its sensitivity, depends linearly on the frequency. However, due to the internal resistance $R_{p}$ and self-capacitance $C_{p}$ of the real sensor, the dependency on the frequency of the voltage difference measured at the coil end $V_{out}\left(f\right)$ is more complex. In the case of a ferromagnetic-core inductive sensor, the frequency response is further complicated by the non-linearity of its self-inductance and sensitivity, which would depend on the magnetic field amplitude as well.

**Figure 2:**
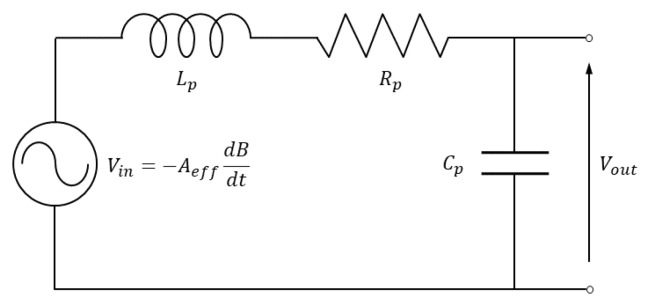
Equivalent circuit of induction sensor with coil self-inductance $L_{p}$, resistance $R_{p}$ and capacitance $C_{p}$.

The frequency response of a real magnetic probe already in a frequency range up to few tens of kHz cannot be reduced to a simple first-order transfer function. An equivalent circuit able to reproduce a typical measured frequency response is sketched in Figure 2, where $A_{eff}$ is the winding effective area. The ratio between the output voltage $V_{out}$ and the time derivative of the magnetic flux crossing the winding $V_{in}=-A_{eff}dB/dt$ describes the non-ideal response of the sensor, relating the actual sensor sensitivity to that of the ideal case as a function of the frequency *f*. This transfer function is given by

(5) $$H=\frac{V_{out}}{V_{in}}=\frac{1}{1+i\omega R_{p}C_{p}-\omega^{2}L_{p}C_{p}}.$$

This transfer function displays a resonance at $\omega_{p}=1/\sqrt{L_{p}C_{p}}$ for small values of $R_{p}$, which limits the high frequency band of the probe.

**Figure 3:**
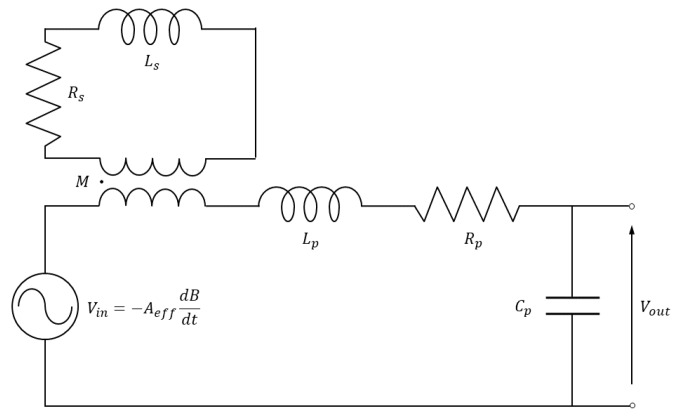
Equivalent circuit of induction sensor with coil self-inductance $L_{p}$, resistance $R_{p}$, and capacitance $C_{p}$, shield self-inductance $L_{s}$, resistance $R_{s}$ and coupling coefficient *M*.

The shielding effect introduced by a potential protective conducting container enclosing the coil or by the cable sheath when the coil is wound with a coaxial cable can be modeled by a magnetically coupled circuit with inductance $L_{s}$ and resistance $R_{s}$, as sketched in Figure 3. In this general case, the transfer function results in

(6) $$H=\frac{1}{1+i\omega \left[\left(L_{s}/R_{s}\right)+R_{p}C_{p}\right]-\omega^{2}R_{p}C_{p}\left[\left(L_{p}/R_{p}\right)+\left(L_{s}/R_{s}\right)\right]-i\omega^{3}L_{p}C_{p}\left(L_{s}/R_{s}\right)\left(1-M^{2}\right)}.$$

where a coupling coefficient *M* between the coil and the shield (close to unity for coaxial cable coils) is considered.

By neglecting the self inductance $L_{p}$ and parasitic capacitance $C_{p}$, the transfer function in Equation (6) is reduced to

(7) $$H=\frac{1}{1+i\omega L_{s}/R_{s}}.$$

showing that, in the case a magnetic shield is adopted, the bandwidth is further limited below $\omega_{s}=R_{s}/L_{s}$.

**Figure 4:**
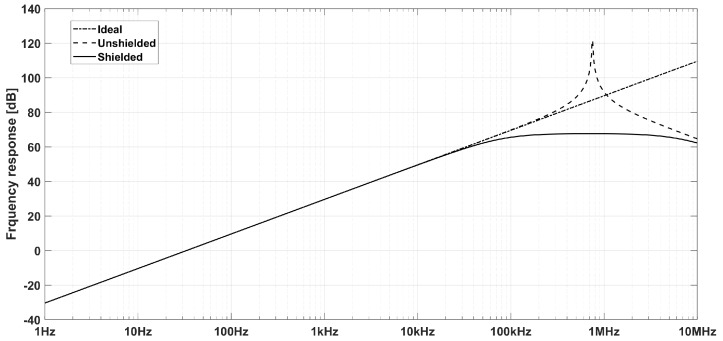
Frequency response of an ideal inductive sensor (dash-dotted), a real one without shielding (dashed) and a shielded one (continuous).

The frequency response amplitude of an ideal and a real (shielded and non-shielded) inductive sensor is presented in Figure 4.

**Figure 5:**
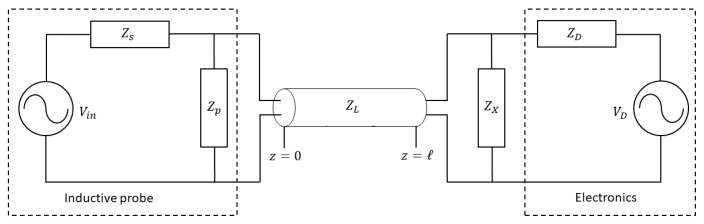
Model of the transmission line along with the magnetic probe and matching impedance.

The above is valid for an ideal measurement of the voltage on an open-ended coil sensor or for the voltage measurement across an infinitely large impedance put between the coil terminals. Instead, in real application, no matter how large the input impedance of the amplifier or digitizer that follows the coil, this will inevitably be of finite value. Besides, as clarified below, the sensor coil is often loaded by a terminating impedance (see Figure 5) which is normally smaller than the electronics input impedance, connected in parallel to improve the frequency response.

### 2.2. Transmission Line Model

The transmission line generally connects the probes directly to the electronics input stage. It usually consists of a thin twisted pair cable, possibly with individual pair shielding. The line has a variable length which can be of some tens to about a hundred meters and has a characteristic impedance on the order of a hundred ohms. As already mentioned, due to the harsh operating conditions the probes undergo inside the vacuum chamber, different cable technologies are adopted for the cable sections internal and external to the chamber, usually characterized by different electrical properties. In some cases the distance between the chamber and the processing unit may be covered with multiple cables as well, again with various electrical properties. Generally, at high frequencies the inhomogeneity of the cables might become important due to wave propagation and reflection effects.

As sketched in Figure 5, the line starts at z=0 on the termination of the magnetic probe which is represented by an ideal voltage source $V_{in}$ with series and parallel impedances equal to $Z_{s}=R_{p}+i\omega L_{p}$ and $Z_{p}=1/i\omega C_{p}$, respectively. As introduced in Section 2.1, at z=ℓ, the line terminates with an arbitrary impedance $Z_{X}$ connected in parallel to a high-impedance data processor represented by its Thévenin equivalent circuit, given by an ideal voltage source $V_{D}$ in series with an impedance $Z_{D}$. The inclusion of the termination impedance $Z_{X}$ allows to adjust the boundary conditions for the wave propagation at the data acquisition input end.

The characteristic transmission line impedance is expressed by

(8) $$Z_{L}=\frac{Z_{0}}{\pi \sqrt{\epsilon_{r}}}acosh\left(\frac{\ell }{d}\right)$$

where $Z_{0}$ is the characteristic impedance of the free space (≈376.73Ω), $\epsilon_{r}$ is the dielectric constant of the cable insulation, *ℓ* is the distance between the conductors and *d* is the diameter of the conductor. An alternative formula is based on the knowledge of the inductance and capacitance of the line:

(9) $$Z_{L}=\sqrt{\frac{L_{l}}{C_{l}}}.$$

As already stated, typical values are $Z_{L}=50÷120\;\Omega$.

Assuming a frequency-invariant transmission line per unit length inductance L, capacitance C, series resistance R and parallel conductance G, the characteristic impedance of the line is $Z_{L}=\sqrt{\left(R+i\omega L)/(G+i\omega C\right)}$ and the propagation constant of the wave $\gamma =\sqrt{\left(R+i\omega L)(G+i\omega C\right)}$. In the case of a short enough line of length ℓ≪1/|γ|, the effect of the transmission line is negligible and in the limit $Z_{c}=1/i\omega C\ell \gg 1$ reduces to that of a parallel capacitance [11]. The analysis of the transmission line is considerably simplified by considering a lumped-circuit model, which means to neglect propagation delays. The approximation is valid for a lossy but distortion-less transmission line terminated at the z=ℓ on a real impedance. The condition for a transmission line to be distortion-less is R/L=G/C [15] and this implies frequency independent attenuation and phase speed of an injected signal. For a given line, of course lumped-circuit and transmission line models discrepancy increases with the frequency, but in general the acceptable level of discrepancy will depend on the context. For example, if only phase information is required at multiple spatial locations and fluctuation magnitude playing no role, a higher level of discrepancy is allowed with respect to an application where instead there are multiple sinusoidal fluctuations at a given frequency and thus measured fluctuation amplitude is significant.

**Figure 6:**
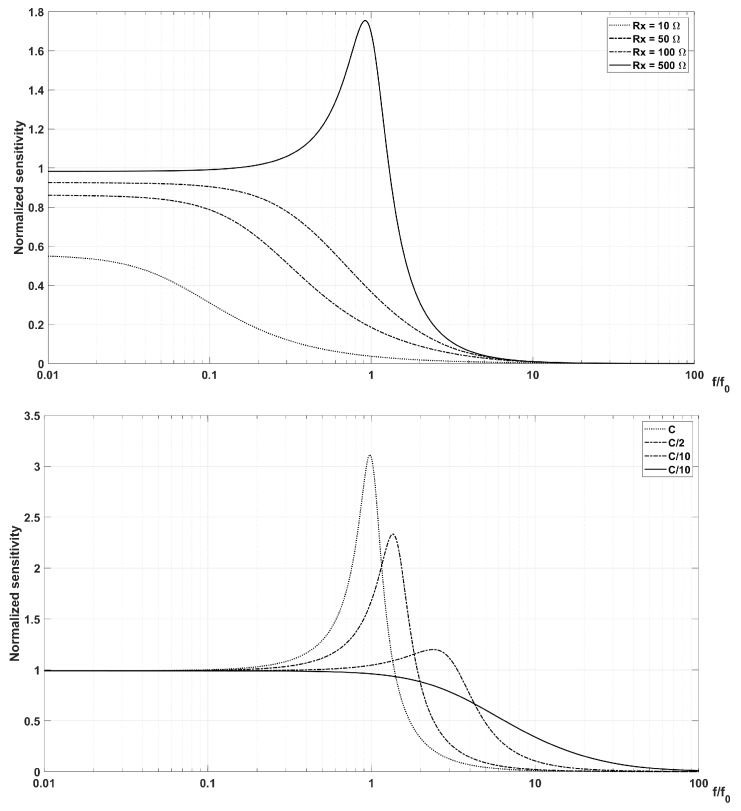
Variation of the frequency response, in terms of normalized sensitivity, with termination resistance $R_{X}$ (**top**) and total capacitance *C* (**bottom**).

Therefore, by considering purely resistive terminating and electronics impedance $R_{X}$ and $R_{D}$, respectively, the sensor circuit results closed by $R_{T}=\frac{R_{X}R_{D}}{R_{X}+R_{D}}$. Consequently, considering for the sake of simplicity a non-shielded sensor coil and the lumped-circuit model for the transmission line, the amplitude $|H^{*}|$ and phase θ of the actual frequency response result, respectively [16]:

(10) $$|H^{*}|=\frac{1}{\sqrt{{\left(1+\alpha \right)}^{2}+\left(\beta^{2}+\alpha^{2}/\beta^{2}-2\right)\gamma^{2}+\gamma^{4}}}.$$

(11) $$\theta =\arctan\frac{(\beta +\alpha /\beta )\gamma }{\left(1+\alpha \right)-\gamma^{2}}.$$

where $\alpha =R_{p}/R_{T}$ is the ratio between the coil and the terminating resistances, $\beta =R_{p}\sqrt{C/L_{p}}$ and $\gamma =f/f_{0}=2\pi f\sqrt{L_{p}C}$ are the quality factor and resonance frequency of the $R_{p}L_{p}C$ circuit. respectively. The relation in Equation (10) shows that the higher is α, the lower is the sensitivity, which means that low coil resistance and high input resistance are desirable. The parameter β has to take into account not only the sensor coil equivalent circuit parameters, but also the transmission line capacitance, which should be added as connected in parallel. Of course, this is true only if the resistance and series inductance of the cable are both negligible. In Figure 6, the typical behavior of the frequency response with termination resistance $R_{X}$ and total capacitance *C* are reported on the top and on the bottom, respectively.

Figure 6 shows that the detrimental effects of the resonance below the first cutoff frequency can be minimized by critically damping the resonance with an appropriate termination of the sensor, or better by properly terminating the transmission line.

In general, the effect of the transmission line is to downshift the resonance of the coil from the frequency observed in the absence of the transmission line. For $C_{p}\ll C\ell$, the first cutoff is due to a resonance between the probe inductance and the line capacitance. Unless the system is correctly matched, line resonances will be present and, depending on the choice of terminations, these may occur well below the first self-resonant frequency. In the case that the line is terminated at the data processor with a correctly matched parallel impedance, the resonance will disappear. However, at low frequencies, the magnitude of the signal is significantly reduced (typically by a factor of 2). Therefore, employing a matched termination avoids all line resonances and optimizes the transient response, but to achieve a correct line matching requires to know in advance the transmission line characteristics. Moreover, this may require different channels with different amplifier gains for the low and the high frequency bands of the signal. Even though a matched termination is ideally the solution for the frequency band limitation introduced by the connecting line, by eliminating line resonances and reducing the time required by the system to reach steady state, it can be inappropriate when system components exhibit significant temperature-dependent resistance.

### 2.3. Filtering and Amplification Stage Design

When the probe and the connections are defined, a matching/filtering circuit must be designed to guarantee the minimum attenuation for the acquisition band signals and, on the contrary, the maximum attenuation for the resonance peaks of the probe and for the higher frequency out-of-band signals. The simplest solution would be loading the line with a resistance of value equal to that of the characteristic line impedance (i.e., impedance of Figure 5 $Z_{p}=Z_{L}$). In this way, the line would of course be adapted, but the acquired signal would appear significantly attenuated. Given the uncertainties on the resistance values of each individual probe and of the lines because of the different lengths for each probe, such kind of termination creates a voltage divider with an unpredictable value. The resulting attenuation should in any case be calibrated probe by probe, with an excessive burden of work and an unreliable result since it would be variable with the temperature.

A more appropriate solution is a termination of some kΩ, which would reduce the resonance amplitude, with an acceptable attenuation of the band signal, lower than the inherent measurement precision (typically, lower than 0.05%). In this way, the variations and uncertainties of resistance would also produce a negligible effect on the signal level.

The best solution in any case is a snubber circuit made up of a capacitor ($C_{sn}$) in series with a resistor ($R_{sn}$), in order to show a high impedance with respect to low frequencies, thus avoiding to dump the signal, and an impedance equal to the resistance alone at high frequency. The snubber cutoff frequency is:

(12) $$f_{c}=\sqrt{\frac{1}{2\pi R_{sn}C_{sn}}}.$$

**Figure 7:**
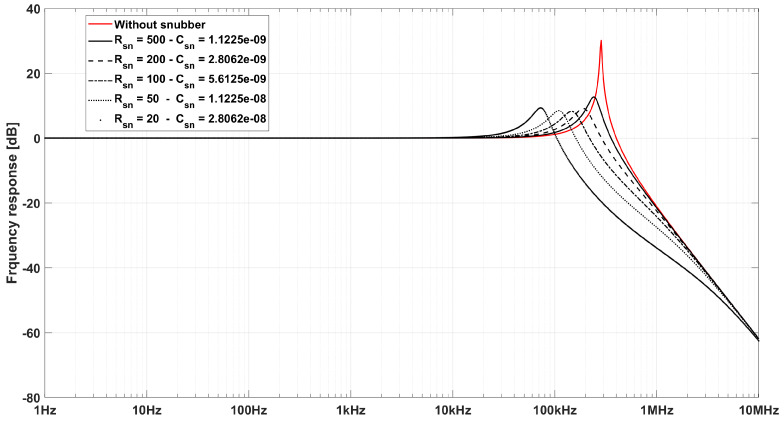
Frequency response of a coil-line system without any filter (red) and different snubber filter (black) with different $R_{sn}$ and $C_{sn}$ parameters for the same cut-off frequency.

The effect of a snubber circuit in reducing the resonance peak is shown in Figure 7 along with that of the $R_{sn}$ and $C_{sn}$ parameters in dumping the peak and determining the new system resonance frequency, for the same snubber cut-off frequency.

Generally, the signal after been passed through a first-order low-pass filter is then compensated in an amplifier stage, usually with programmable gain. Evidently, a high common mode rejection of the amplifier is required in order to not deteriorate the measurement sensitivity. An automatic compensation of the global offset and a saturation detection are useful solutions to improve the precision and reliability of the measurement, respectively.

Amplification of signals detected by magnetic probes needs particular care because of their typical wide bandwidth and large amplitude fluctuation. Typically, the low-frequency signal components are on the order of 10 mV, while already at some tens of kHz signals can be of the order of 10 V signals. In addition, sudden disruptive fast MHD events can produce spikes up to several hundreds of volts. Therefore, to prevent saturation of the amplification stages but maintain a reasonable signal level, it can be beneficial to have a pre-amplifier preceded by a typically first-order passive filter with a cut-off pole around 100 Hz and a zero at a few kHz. The transfer function of this filter has to be chosen in order to give a global transfer function of a perfect integrator when combined with the input stage filter of the integrator and the integrating stage itself.

### 2.4. Attenuator Design

A voltage divider or buffer amplifier at the end of the transmission line is used to match the electronics input voltage and impedance. The voltage divider should have an input impedance to match the impedance of the coil-transmission L-C circuit and an output resistance to match the electronics impedance (e.g., 50 Ω), in order to present a flat response up to the L-C resonance. An additional capacitor is placed in parallel with the non-acquired resistor, so as to further filter the signal by draining any remaining spurious high frequency voltage peaks away from the integrator input. An active amplifier with the proper input and output impedances may be alternatively used for the same task.

### 2.5. Uncertainties and Sensor Precision

The typical required precision from magnetic field probe measurement is a fraction of 1%, and to reach this each element in the signal acquisition chain must be calibrated with a precision on the order of 0.1% [11]. Usually, manufacturing of an inductive probe consists of winding several layers of relatively large wire compared to the small probe coil, which results in a poorly reproducible mechanical process. For this reason, the effective area of the probe cannot be estimated with the required precision and therefore the area of each probe has to be calibrated one by one. This is done by inserting the probe in a region of a solenoid or Helmoltz coil where the produced magnetic field is precisely known to be very uniform [14,17]. In a similar way, the cross talk with respect to perpendicular magnetic field is also measured with the same equipment. The transfer function between the current generating the magnetic field and the voltage on the probe is measured in a range between a few Hz and several kHz. This function is then extrapolated also to 0 Hz and used to derive the probe area. To perform a precise characterization of the probe effective area, the measured data points have to be fitted to at least a second-order transfer function of the form

(13) $$H=\frac{1}{\left(1+i\omega \tau_{1}\right)\left(1+i\omega \tau_{2}\right)}.$$

using an algorithm, for example the one described in [18].

Differences in the acquisition path from the probe to the electronics among the different channels must be known or minimized. In situ measurement of each impedance over the frequency range of interest can be achieved by injecting a voltage signal at the cable end and measuring the driven current [11]. This allows taking into account with a single measurement the intrinsic variations among the electromagnetic characteristics of different channels due to the single winding, cabling and possible shielding.

## 3. Analog vs. Digital Integration

**Figure 8:**
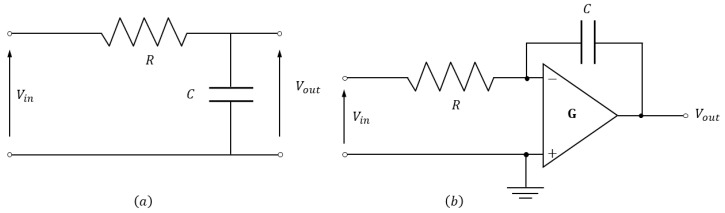
Passive (**a**) and active (**b**) integrator circuits. The active circuit uses an operational amplifier with gain G.

As mentioned in Section 2, to obtain a signal proportional to the flux linked by an inductive sensor, the raw voltage signal from the sensor must be integrated. A simple RC circuit, as shown in Figure 8a, provides a good approximation of an ideal integrator for high-frequency signals. The transfer function between the output and input voltage of the integrator circuit is

(14) $$V_{out}=\frac{1}{1+i\omega \tau }V_{in}\approx \frac{1}{\tau }\int V_{in}dt\;\;\;\;\left(\omega \tau \gg 1\right).$$

where τ=RC is the characteristic integration time. This approximation is valid for timescales lower than τ. The relation in Equation (14) shows that, unfortunately, the attempt of extending the allowed integration timescale by increasing τ would necessarily cause the unfavourable result of reducing the output voltage.

The conflicting limitation on integration timescale and signal amplitude can be greatly extended by the use of active integrator circuits. The most basic circuit uses an operational amplifier to perform the active integration, as shown in Figure 8b. The output voltage is given by

(15) $$V_{out}=-\frac{G}{1+i\omega \tau +Gi\omega \tau }V_{in}\approx -\frac{1}{\tau }\int V_{in}dt\;\;\;\;\left(G\omega \tau \gg 1\right),$$

where *G* is the open-loop gain of the operational amplifier. The relationship between $V_{out}$ and $V_{in}$ depends again on τ=RC as before, with the advantage in this case of an integration timescale increased from ∼RC to ∼GRC, where *G* can be on the order of $10^{5}÷10^{6}$. Therefore, by adopting *RC* values between 1 ms and 1 s, this allows the integration of sensor signals in experiments with typical pulse lengths up to 10 s.

The primary issue of active integrator design and operation is the so called integrator drift. Any small differential DC offset on the integrator input introduced by the active electronic components is added to the sensor signal and integrated along with it. The resulting output is distorted by an error term which increases at least linearly with time. It is clear that this problem becomes particularly significant when real-time measurements are needed in experiments requiring long lasting pulses. The elimination or mitigation of such input offsets with a high degree of precision and reliability is therefore essential.

Drift is introduced by both systematic offset or random noise in the integrator input voltage. Particularly detrimental in typical application is the flicker 1/f noise [19]. The main voltage offset sources are:

- intrinsic imperfections of the electronics, such as the amplifier’s input offset voltage and bias currents, and imbalance of discrete and integrated circuital components;
- electromagnetic interference rectified by non-linear components;
- thermoelectric voltages along the acquisition chain;
- capacitors leakage that directly impacts the measurement value.

In addition, some of these typically increase in time and therefore the longer is the pulse, the greater is the rate of drift divergence (becoming greater than linear).

### 3.1. Integrator Concepts

First, a careful choice of the input electronics is required to mitigate the root of the problem. Then, different strategies and precautions can be adopted. In particular, there are three state-of-the-art integrator concepts developed for magnetic field measurements for real-time control purposes:

1. Analog integrators based on a pre-compensation circuit: The output voltage is measured before each pulse during a time interval when no excitation is applied, sampled and held by analog or digital means, and then looped back to the amplifier during the pulse [20,21].
2. Analog or hybrid analog–digital implementations measure both the signal and the drift, alternatively [22,23] or continuously [24], and correct the measured signal many times during the pulse.
3. The measured signal is chopped early in the data acquisition chain and the digital de-chopping operation ideally removes any DC offset voltages introduced anywhere along the measurement chain [25,26,27].

Both the last two techniques imply an expensive design and a complex implementation that are not compatible with a very large number of sensors to be integrated. In addition, only offset sources internal to the integrator are taken into account, while external ones remain in the signal.

#### 3.1.1. Analog Integration

A feedback circuit that adjusts the input offset to minimize the output voltage is a straightforward means for compensating the drift error. The feedback circuit is activated between experiments, when the sensor gives no significant signal, while it is disabled during the experiment and the latest measured offset is maintained by an analog or digital sample-and-hold circuit. However, this is based on two assumptions: the temperature in the integrator rack does not change significantly during the time of an experiment, so that the compensation value held before the start is relevant for the entire pulse duration, and the compensation value measured before the pulse itself remains stable during the pulse. This kind of integrator has proved to be able to achieve output drifts of few mV in 1000-s pulses [20,21]. Such a low level of drift was achieved by the use of operational amplifiers with low input bias current, with low voltage offset (few μV) and their voltage offset stability (few tens of nV/°C), and the use of expensive high quality low-leakage polypropylene capacitors both in the integrator and in the compensation voltage holding circuit, which have a very low equivalent serial resistance (ESR) and dielectric absorption. Another solution is the use of a digital memory instead of the capacitor, in association with an ADC and a DAC converter. In addition, the common mode rejection ratio (CMRR) of the integrator is improved by implementing the galvanic insulation of the signal and the power supply. However, analog integrator designs of this kind are inherently limited in pulse length by the level of technological development of electronic components and by possible fluctuations of the offset during a long discharge, which indeed should be estimated as often as possible.

#### 3.1.2. Digital Integration

Several schemes adopting the digital technology have been implemented, hybrid solutions in combination with analog circuits or pure digital implementations, in order to go beyond the analog limitations. Analog integration with a digital feedback circuit gets rid of the changes in the compensation caused by capacitor leakage current over time. For instance, in [22], two analog integrated signal are used for the compensation of the drift through the digital combination of the two signals. One analog integrator processes the sensor input signal, while the other is connected to a dummy load with a resistance equal to the sensor loop and cables, which allows the measure of the drift. Digital signal processing combines the two integrator outputs into a single real-time digital signal, thus corrected from the measured drift. Furthermore, the two integrators periodically reverse roles in order to eliminate on average the differences between the two. In integrators of this design, the drift error is reduced, and it is no longer linked to the pulse length because its correction is based on a measurement updated many times during the pulse. The droop error during constant magnetic field interval is reduced as well by digital compensation of the recorded value at the end of the previous transient phase.

The integrator droop is instead completely eliminated by the full digital integration. In the basic scheme, the input is periodically sampled and the digitized signal is then numerically integrated. However, digitization of the raw sensor signal has drawbacks as well. Indeed, new errors are introduced by the finite time resolution, the finite bit resolution and the voltage saturation of the ADC. Significant errors can result in the integrated signal during transient events with timescales comparable to the sampling interval and then persist throughout the pulse. Time resolution errors can be mitigated by choosing a sampling rate fast enough with respect to the phenomena to be observed. Voltage saturation of the ADC may also occur in case of fast events which tends to produce large dB/dt. This issue can be reduced by using parallel input channels with different gains and selecting the high-gain channel except when saturated. However, the best option to tackle both the limitations of time resolution and ADC saturation is by using a low-pass (RC) filter in the input to the ADC, for smoothing out the fast transients and reducing their peak voltage. Then, a low bit resolution introduces a significant error, which increases linearly with time in the case of constant signal. In this respect, the presence of few bits of noise is beneficial since the input error becomes no longer systematic but random and the integral error is much smaller.

#### 3.1.3. Chopped Digital Integration

To, in principle, completely eliminate the integrator drift, the ADC input can be rapidly chopped at high frequency alternating between the filtered sensor loop and a matched dummy load [23] or between the two polarities of the filtered sensor loop [25,26,27]. In these integrator schemes, both the sensor signal and any offset voltage generated within the integrator are measured during the integration and the latter is then continuously corrected using an offset value which is update each chopping period.

If the errors introduced by the finite resolution of the ADC are measured and corrected periodically during the pulse, they can be assumed uncorrelated from one measurement cycle to the next. When integrated, the resulting accumulated error is proportional to $\sqrt{N}$, where *N* is the cycle number. At a fixed sampling rate, this means a drift error growing with $\sqrt{t}$, as confirmed in measurements [23]. A digital scheme implementing error correction based on offset measurement only at the beginning of the pulse will likely present drift error which instead increases at least linearly with time. Therefore, for very long pulse application methods implementing a periodic correction of the input offset must be chosen in the case of digital integration design.

It is clear that digital technology schemes can have several advantages, in particular related to long lasting applications, but an accurate digital integration of the coil sensor signal requires a careful choice of the integration period, sampling frequency and triggering time, especially in the presence of complex and unknown frequency content in the processed signal. Moreover, good quality digital data acquisition (DAQ) components are typically very expensive when high dynamic, wide bandwidth and high precision are required. Furthermore, the injection of high-frequency noise from the chopping, as well as the loss of bandwidth and phase lag due to the necessary filtering affect this integration method.

### 3.2. Noise in the Acquisition Chain

Ensuring the highest signal to-noise ratio of the acquired signal is essential in order to perform correct integration. Noise is an unwanted signal disturbance that can cause important variations in the acquired data, even making them completely unusable. In particular, it is a critical parameter in the integration process since, in carrying out the operation, its effects add up and makes an important distortion of the resulting signal, especially if the acquisition continues for several seconds. Many sources of noise affect the signal from a pickup coil, such as magnetic and electrostatic pickup from imperfect shielding, inductive common mode signals from possible extended cabling, electronic noise, thermal noise, radiation induced electromotive force (RIEMF), etc. These generally add up in a background incoherent white noise, which is a random signal having equal intensity at almost all frequencies, therefore having constant power spectral density. The 1/f noise (or flicker noise) instead has a power spectral density inversely proportional to the frequency of the signal and almost all electronic devices are affected by such kinds of noise. The system is therefore affected at low frequencies by flicker 1/f noise and for subsequent frequencies by a white noise.

**Figure 9:**
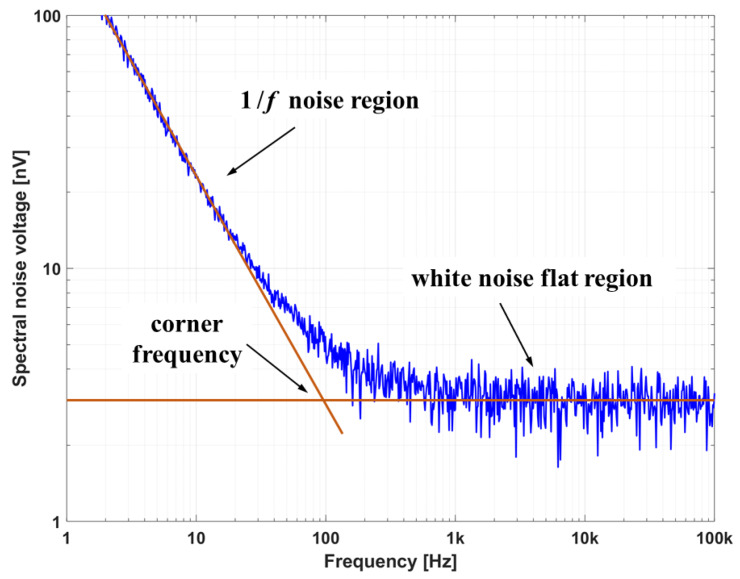
Typical noise in a differential amplifier.

In Figure 9, the typical spectra of a differential amplifier is shown. Flicker disturbance is particularly harmful and difficult to eliminate even with post acquisition processing because there is no simple relationship (as in the case of white noise) that links the noise level to the standard deviation of the integrated signal and, when the signal is integrated, it translates into final drift errors which increase at least linearly with time. It is therefore essential in the DAQ design to carefully choose electronic components which guarantee high performance especially in terms of noise level. However ADC manufactures usually do not provide adequate information related to the flicker in specifications and thus the devices require adequate custom testing before being adopted.

## 4. RFX-Mod2 Pick Up Coil System

Magnetic confined fusion machines require real-time feedback control systems characterized by a response time of the order of 1 ms for both protection needs and the study of fast physics phenomena. RFX-mod stands out among other magnetically confined plasma machines for its advanced and flexible systems for active feedback MHD control, based on a network of 192 independently driven saddle coils (4 poloidal × 48 toroidal), a state-of-the-art digital real-time control system and a comprehensive set of diagnostics. This makes RFX-mod an excellent test bench where challenging regimes in both Reversed Filed Pinch (RFP) and Tokamak configurations can be achieved. RFP plasmas with a dominant helical shape, opening a natural connection to the stellarator geometry, and stable low q Tokamak plasmas have been obtained thanks to the interplay of the extended magnetic measurement system and the non-axisymmetric coils in counteracting the growth of the plasma instability. The innovative and powerful feature of RFX-mod is the capability of operating under challenging boundary conditions: many input signals, many modes to control and fast response required.

**Figure 10:**
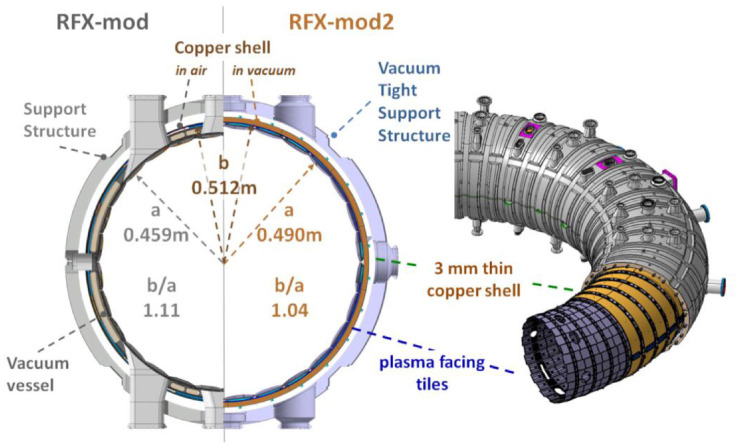
RFX-mod2 toroidal assembly, poloidal cross section and isometric view.

As anticipated in previous paragraphs, an upgrade of RFX-mod presently being implemented foresees a modification of the machine assembly (see Figure 10) based on the present understanding of the interaction between passive conductive boundaries and tearing modes, which should improve the RFP confinement and allow the investigation of a broad spectrum of plasma physics.

**Figure 11:**
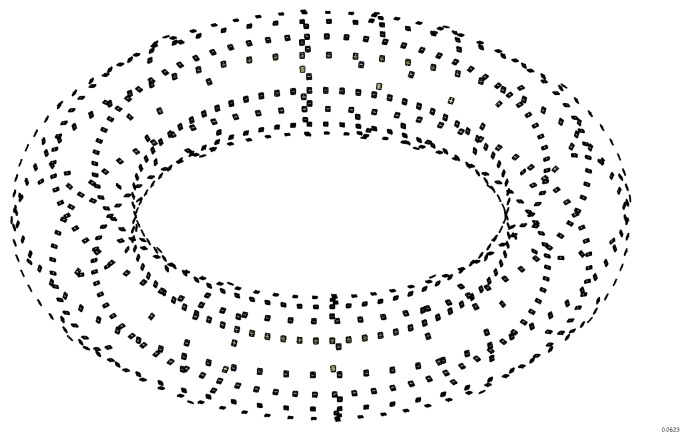
Layout of the 8 toroidal and 13 poloidal arrays of pick-up coil sensors.

The RFX-mod2 assembly modifications are an opportunity for the complete renewal and improvement of the magnetic diagnostic system, from the probes moved inside the vacuum vessel to the integrator modules. The greatly increased number of magnetic sensors (724 pick-up probes for 1088 signals, see Figure 11) will allow an improved characterization of the spatial harmonic structure of the plasma in both RFP and Tokamak configurations, as well as perform a more precise removal of sidebands aliased in the measurement signals due to the finite number of active coils.

In this paragraph, the design of the new system as a whole and the tests on the performances of the digital interface applied to a prototype of the new pick-up probe are described. In our application, a scheme implementing purely numerical integration is adopted without chopping, given the typical duration of a pulse of a few tens of seconds, which allows a compact and cost effective system for the integration and processing of the about 1700 signals from inductive coils and loops. Therefore, the main elements constituting the system are described, focusing especially on those components which are critical in determining a low enough noise level.

### 4.1. Magnetic Probe and Connecting Line Characterization

**Figure 12:**
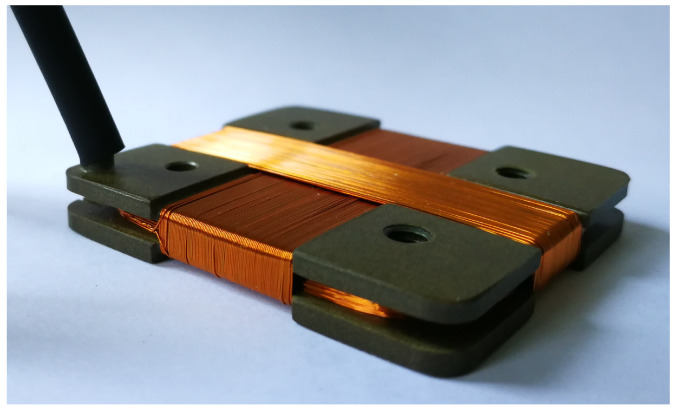
Prototype of a three axis pick-up magnetic probe for RFX-mod2 (37 × 42 × 5 mm).

The new probes of RFX-mod2 (see Figure 12) are three axis pick-up coils, wrapped with grade 2 class C AWG 33 enamelled copper wire, with effective sensing area of about 250 cm${}^{2}$ for those measuring the field component tangential to the plasma boundary and 2000 cm${}^{2}$ for those measuring the radial component, which offer a good compromise between compactness, signal sensitivity and usable bandwidth. They are manufactured with materials and processes suitable to comply with the vacuum installation [10].

**Figure 13:**
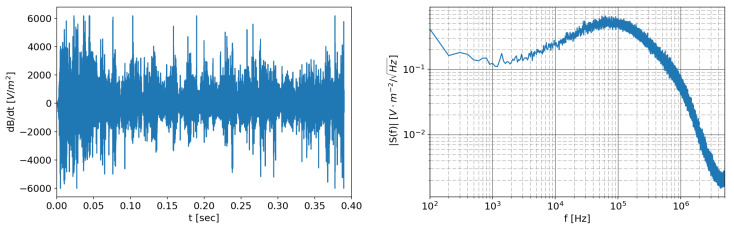
dB/dt signal of the toroidal field component from an in-vessel wide bandwidth probe from a reversed field pinch pulse at 1.5 MA in RFXmod: (**left**) signal; and (**right**) spectrum.

The maximum level of the signals that will be acquired was derived from experimental measurements already carried out on RFX-mod, which can be used to estimate the level of the signal once the probe characteristics and its measurement chain are defined. An example of the expected dB/dt signal source and its spectrum, obtained by wide-band in-vessel probes installed on RFX-mod, are shown in Figure 13. This signal will be later used to set the maximum signal level.

On the other hand, the minimum signal requirement involves the sensitivity to slowly changing magnetic field at low intensity. Magnetized plasmas are prone to amplify the spatial resonant component of an external field. Even small unwanted field variations (so-called “error fields”) can have large effects on the plasma behavior. The ability to detect these features is thus needed for example to allow the characterization of the structure of these small error field components and the correction of misalignments of the sensors.

**Figure 14:**
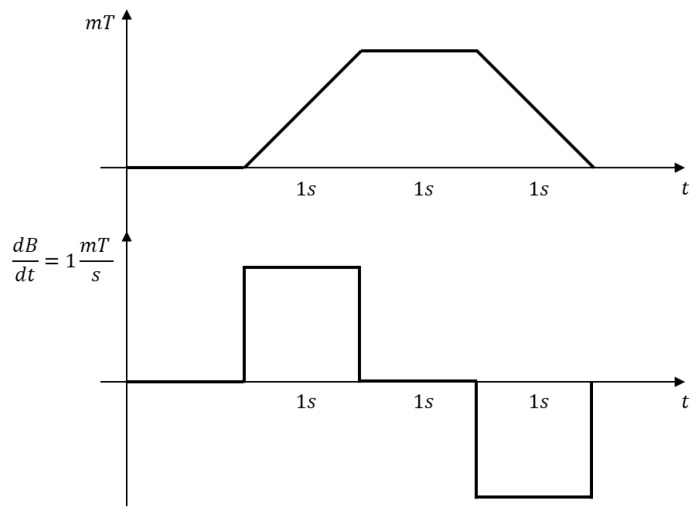
Desired minimum detectable signal scenario.

As the minimum detectable reference signal, we choose a trapezoidal 1 mT field with a slow trapezoidal evolution: 1 s ramp-up, 1 s steady state and 1 s ramp-down (Figure 14).

To build a model of the acquisition chain, the electrical parameters of a coil prototype (very similar to other already tested), as shown in Figure 12, were taken into account. The three windings of the prototypes have been characterized in terms of effective area and cross-talk pick-up inside a lab solenoid producing a uniform magnetic field with a field-excitation current transfer function known with high accuracy. The main results are shown in Table 1.

**Figure 15:**
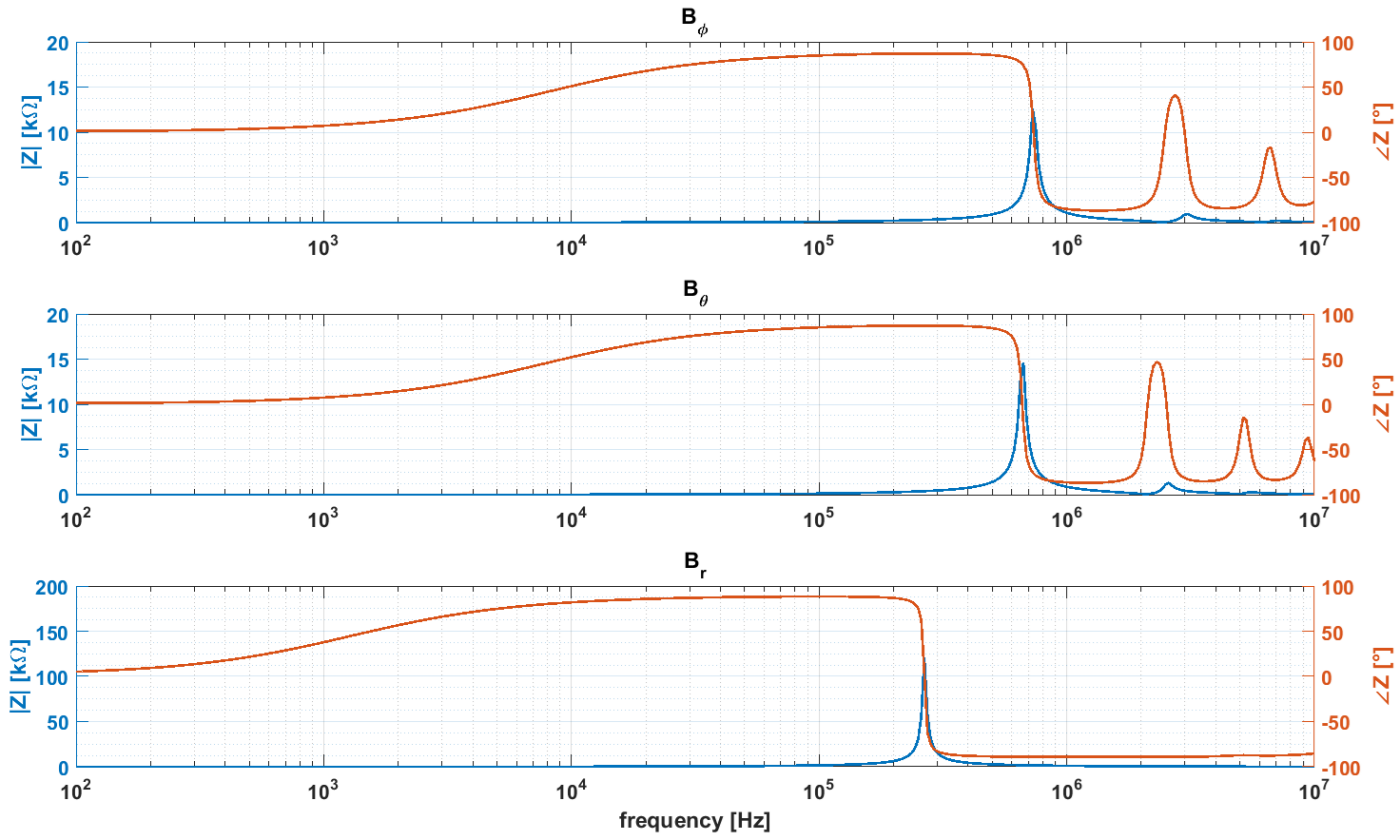
Frequency response in module (blue) and phase (red) of the impedance of the three prototype windings.

The three coils of the prototypes have been tested with an impedance analyzer HP4198A by 400 automatic measurements of the impedance between 100 Hz and 10 MHz. The measured parameters of the equivalent RLC circuit for the three windings are reported in Table 2 and the impedance frequency response is shown in Figure 15.

The twisted coil cable is used without transition up to the vacuum feed-through, covering a distance between 2 and 3 m. A characteristic impedance between 150 and 200 Ω has been estimated in the two cases. The twisted pair uniform transmission line connects the probes directly to the anti-aliasing filter. The cable used is an AWG 24 insulated with LSZH technopolymer, with a dielectric constant of 3.2. The line has a variable length between 20 and 30 m and has a characteristic impedance of about 98 ohm.

A passive snubber filter with two branches and related cut-off frequencies $f_{sn1}\approx 1.6$ MHz and $f_{sn2}\approx 16$ kHz, respectively, has been designed to remove the main resonance peak from the coil-line frequency response and to smooth out the residual high frequency peaks.

A passive attenuator has been adopted to adapt the output voltage from the filter to the ADC input. It consists of a simple resistive divider with the addition of a capacitor in parallel to the grounded resistance in order to filter out the remaining spurious voltage peaks. The resistor values have been chosen according to the ADC dynamic range, considering the requirement of being able to distinguish 1 mT/s which translate to 0.02 mV with a 200 cm${}^{2}$ probe effective area.

### 4.2. Analog Integrator Reference Characteristics

**Figure 16:**
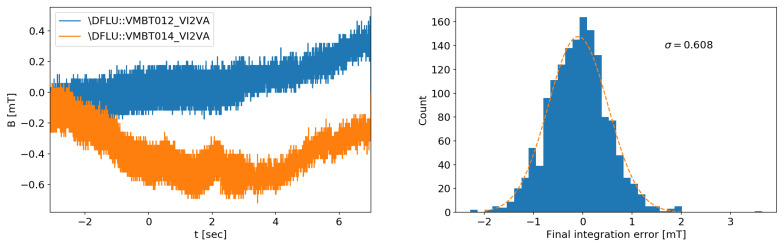
Examples of integrated signals (**left**) and distribution of final integration error of the analog integrators of RFX-mod after 10 s, from toroidal field sensors on eight “dry” pulses (1510 signals (**right**).

As a guiding reference case, the performance in terms of noise level and integrator drift of the existing conditioning system of RFX-mod [28] is shown in Figure 16. The integration gives a final error on the order of 0.6 mT. The signal range to be measured spans from 10 mT to 0.5 T. Even if most of the integration drift error can be corrected in post-processing, it is still necessary to keep it into an acceptable range to allow the use of these signals for real-time control purposes.

### 4.3. ADC Noise Characteristic

**Figure 17:**
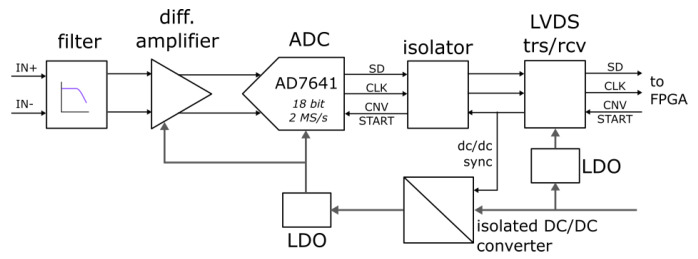
Block schematic of the ADC module.

The noise characteristic of a precision ADC (AD7641, 2 MSamples/s, 18-bit SAR ADC) has been measured by taking advantage of the existing modules of the ATCA-MIMO system [29,30]; the basic structure of the module is reported in Figure 17. For the subsequent test shown, the input stage has been disconnected and the reported test have been performed with both the on board synchronous DC/DC converter and with a separate linear power supply.

The module has been interfaced with a readily available Red Pitaya board [31], preparing a custom configuration for its Xilinx Zynq field-programmable gate array (FPGA), in order to investigate the capability of this specific architecture (see Section 5 for further discussion).

**Figure 18:**
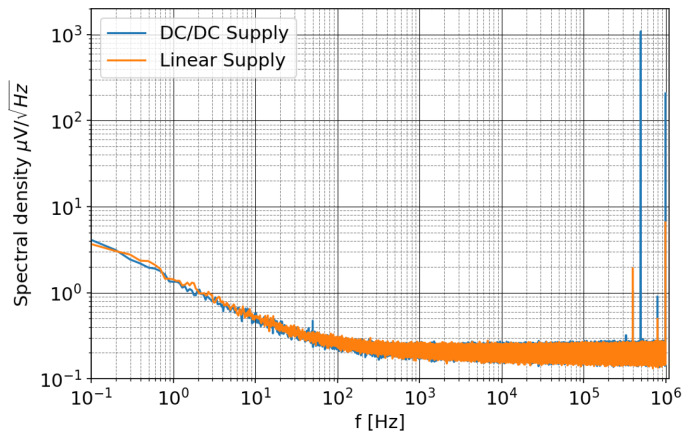
Noise spectral density for the AD7641 with two type of power supplies; the difference is in the level of harmonic associated to the DC/DC converter, while the 1/f noise appears unaffected.

The frequency domain analysis (Figure 18) shows two main peaks due to the isolated DC/DC converter, one at 500 kHz and its 1 MHz harmonic. At low frequency, the spectrum is dominated by the 1/f noise, which appears to be insensitive to the type of power supply used.

### 4.4. Direct Digital Integration Capability

For short time integration, the noise spectrum involved is in the high frequency range, i.e., dominated by the white noise. In this case, the numerical integration of a sampled sequence $s_{i}$ is a well-defined case, being equivalent to a discrete random walk in which the distribution of the steps $s_{i}$ has zero mean with a finite variance $\sigma^{2}$. Thus, given the final integral sum after *n* steps $S_{n}=\sum_{i=0}^{n}s_{i}$, its variance is simply proportional to number of steps: $E|S_{n}^{2}|=\sigma^{2}n$.

**Figure 19:**
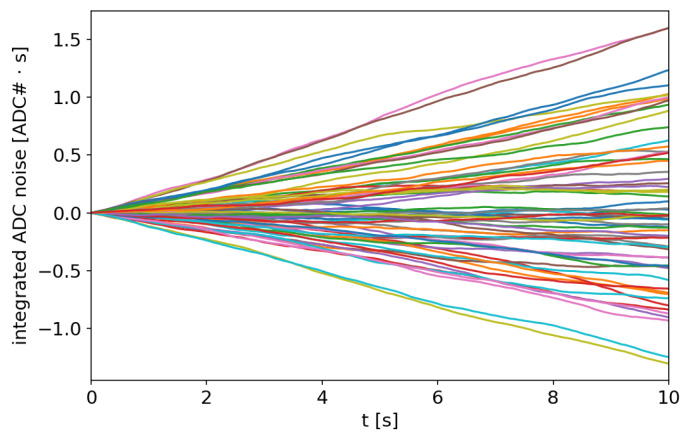
Noise integrated signals from the module with the input amplifier disconnected and fed by the switching DC/DC converter.

**Figure 20:**
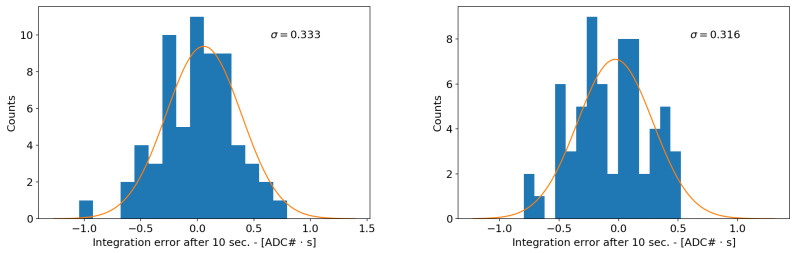
Histograms of the final integration values due to the integrated noise with DC/DC (**left**) and linear power supply (**right**).

In the case of RFX-mod2 signals, the integration time lasts several seconds and the desired frequency range extends well below the typical 1/f corner. The integration is then dominated by the flicker noise level. Contrary to the white noise, for this case, there is no simple relation linking the noise level to the standard deviation of the final integration step. For this reason, the problem has been tackled by means of a statistical analysis based on experimental data. An ensemble of integrated noise is shown in Figure 19 and the distributions of the final integration error are reported in Figure 20. After 10 s, the final integration error has a standard deviation of 0.33 ADC counts·s in the case of the DC/DC power supply, while with the linear one there is a slight improvement to 0.316 counts·s.

**Figure 21:**
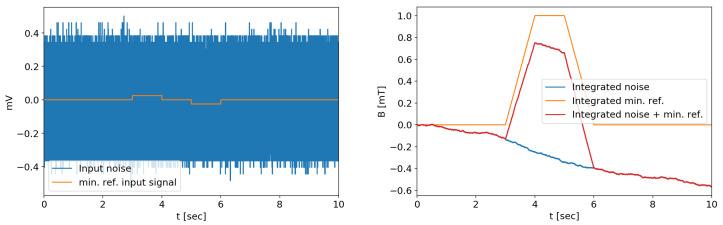
Expected signals with the noise generated solely by the ADC input, with the minimum reference input signal. The integrated signal is clearly visible.

An estimation of the expected output with the reference signal of Figure 14 can be obtained by adding the equivalent input reference signal (Figure 21, left) to a noise sample. After the numerical integration, the desired signal can be successfully identified with an overall acceptable error (Figure 21, right), and it can then be further corrected by suitable post processing, as the integrated error is fairly linear.

### 4.5. Experimental Direct Numeric Integration Test

As a final proof of concept, the complete signal acquisition chain has been deployed in a test bench environment with the actual prototype of the probes. The test was carried out with a solenoid connected to a laboratory power supply. The current on the solenoid was modified by manually changing the power supply settings.

**Figure 22:**
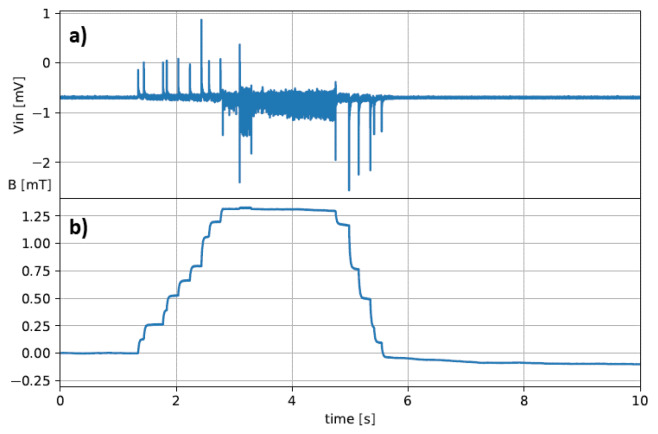
Test of numerical integrated signal from an actual probe with real field applied: direct acquired signal in (**a**) and numerical integration result in (**b**).

The direct acquired signal is reported in Figure 22a and the numerical integration result in Figure 22b, which appears to be very good. Note that the level of the signal in Figure 22 top, is in the very low range of the ADC, with the full signal scale at ±5 V.

## 5. DAQ System Architecture

**Figure 23:**
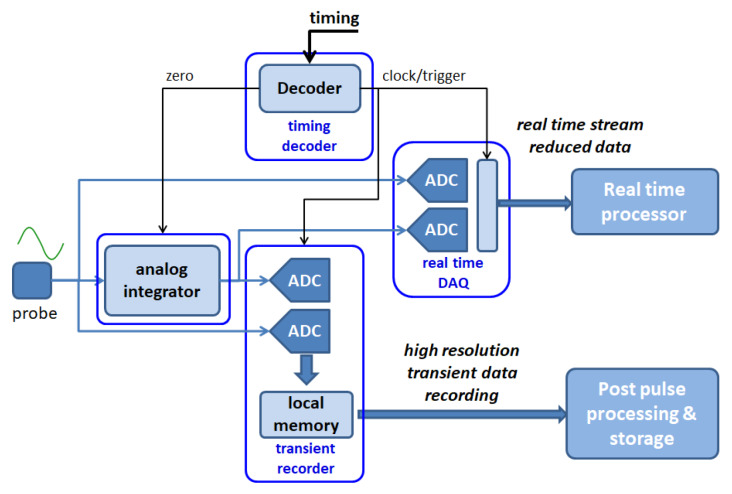
Block scheme of the legacy architecture of RFX-mod. Each function is carried out by a separate dedicated hardware component.

The existing legacy architecture of RFX-mod [28,32], where each function of the system is assigned to a separate hardware block, is reported in Figure 23.

As mentioned in Section 1, the complete replacement of existing analog integrators with direct numerical integration of the digitally acquired signal has been chosen for the new magnetic measurement system of the upgraded RFX-mod2 experiment allowing a significant reduction of the hardware complexity. In principle, for each sensor, this system would require four ADC channels, two for the integrated signal and two for the derivative component, since the channels are duplicated for the high resolution transient data recording and for the real-time control system. Since the foreseen number of channels is quite high (about 1700), the direct digital integration performed numerically offers a dramatic advantage over its analog counterpart in term of compactness, cost, reliability, deployment, calibration and maintenance. Moreover, the architecture is suitable for performing the numeric integration needed for real-time plasma control, while simultaneously recording the dB/dt signals needed for the study of the fast MHD dynamic processes taking place inside the plasma [33,34].

The basic architecture of the proposed system resembles that of the ATCA-MIMO [29], originally developed for the plasma column vertical stabilization of the Joint European Torus (JET) tokamak experiment and already tested on RFX-mod [30]. The acquisition channels of this architecture are isolated input ADC modules coupled to an FPGA which then routes the data flow to the processing/storage servers through a PCI-e interface.

**Figure 24:**
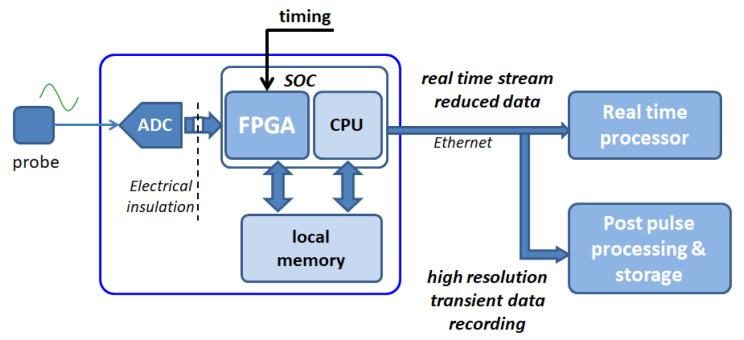
Block scheme of the new DAQ architecture for RFX-mod2, based on a System On Chip SOM. The DAQ board performs all necessary function by itself.

We are planning to switch to a slightly different and more modern architecture, maintaining the basic feature of the single isolated ADC module for each channel, which is expected to highly improve the noise immunity. The system management is performed through a commercially available System-On-Module (SOM, based on the Xilinx Zynq architecture), integrating both FPGA and CPU cores, which allows to set-up a self-contained acquisition 6U eurocard board, managing up to eight channels each (see Figure 24).

This solution easily provides both the autonomous local storage for transient recording function, as well as the ability to send a real-time data stream of decimated and integrated signals to the control servers through standard gigabit Ethernet interface. The use of a configurable FPGA is intended to handle ADC conversion and provide a set of on-line functions directly performed at the FPGA logic level, including the numeric integration in real-time, the recording of the derivative signal dB/dt and the signal decimation for the lower frequency data stream (up to 10 kSamples/s) required by the real-time control system.

The synchronization of the system can be handled directly by extracting the timing information from a variety of the central time sources, such as an external timing highway or by using the PTP protocol through the Ethernet interface.

The time critical functions carried out by the FPGA in this context are:

- the management of a circular data buffer and the DMA transfer in RAM of pre and post trigger samples after the trigger has been received;
- anti-aliasing filtering and subsequent sub-sampling of the samples to be streamed, where the resulting samples are enqueued in a FIFO accessed by the processor;
- digital integration for deriving magnetic field measurements from the coil signals (it is worth noting that in this case a single ADC stage will generate two ADC channels);
- ROI detection in case ADC triggers are derived from the signal itself (e.g., over a given signal level threshold); and
- clock and trigger extraction in the case a highway signal is provided by the timing system, encoding both clock and triggers.

The less critical functions that will be carried out by the processor unit are:

- the management of the configuration setting, received via TCP/IP or HTTP, where the processor validates the configuration and writes the identified registers in the FPGA;
- off-line data readout of acquired samples in transient recording and communication via TCP/IP with the central data acquisition system; and
- network data streaming of sub-sampled data read from the FIFO and sent in UDP packets to the active plasma control system.

**Figure 25:**
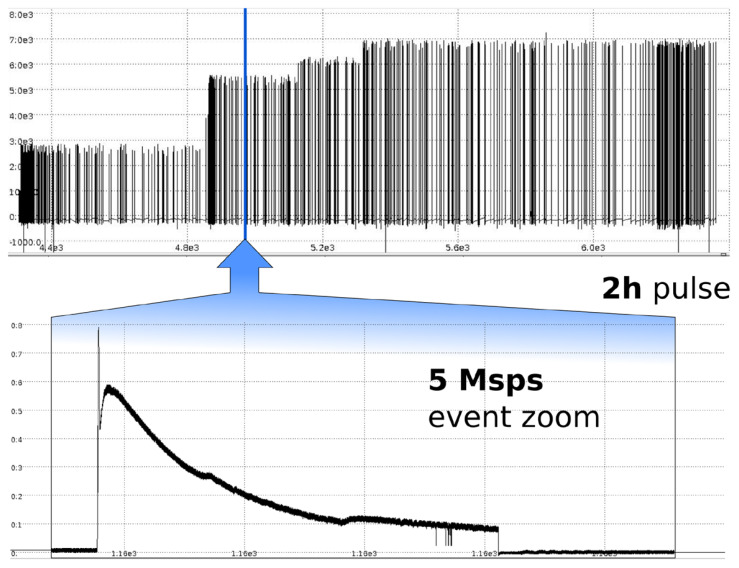
Current measurement on the extraction grid of NIO experiment, with self triggered high- resolution capture of breakdown events.

The flexibility provided by the configurable FPGA also allows the inclusion of more sophisticated triggering mechanisms and a deeper integration with the timing systems. An example is given by the acquisition of fast transients requiring high speed sampling only in a given, dynamic ROI. This technique has been applied to NIO experiment [35], a small radio frequency negative ions beam source with an electrostatic particle accelerator stage composed of several high voltage grids. In this environment, the occurring breakdown events lead to a rapid change in the measured currents and voltages of the grid power supply that need to be measured in detail [36]. To this purpose, a subset of the FPGA functionalities given above and required for magnetic DAQ system had already been implemented on a Red Pitaya device: the trigger logic to detect the occurrence of the event, the pre and post trigger sampling logic and the FIFO/DMA data transfer to computer memory via the GNU Linux driver. This implementation allowed the proper handling of the measurements, as shown in Figure 25.

## 6. Conclusions

Many experimental applications involve the study and/or control of magnetic fields whose range and frequency spectra demand the use of inductive sensors for their characterization. Inductive sensors indeed have the important advantages of robustness, operation and design simplicity, wide frequency bandwidth and large dynamics. However, inductive probes exhibit some shortcomings as well: a probe fixed in space can detect only time varying magnetic fields, although with a frequency down to mHz range, and, since the output signal depends on the time derivative of the magnetic field, it is frequency dependent. This in turn requires the integration of the sensor output with the possible introduction of additional errors in the signal processing and evidently the accuracy of the measured data strongly influences the machine controllability and the scientific results.

The probes of the new magnetic measurement system of the upgraded RFX-mod2 experiment have been moved inside the vacuum vessel, adopting materials suitable for high temperature applications presenting low outgassing rate and similar thermal expansion coefficients for both the winding cable and the reel. The careful design of the coils allowed obtaining a high frequency band despite the stringent limitations in terms of available room. The probe-transmission line system has been characterized and the associated filters and attenuator accordingly designed. The complete replacement of existing analog integrators with direct numerical integration of the digitally acquired signal has been chosen in view of a significant reduction of the hardware complexity.

The architecture of a new flexible ADC device has been presented, aimed at reducing the number of actual ADC channels by integrating high speed transient recording and data streaming for real-time plasma control. In addition, the same ADC will be used to acquire both magnetic fields and their time derivatives, providing FPGA based digital integration for the derivation of the former, providing a significant cost reduction with respect to the duplication of devices for transient recording and streaming.

A complete acquisition chain has been built, testing the frequency response and dynamic range of the system in bench tests. A complete isolated ADC module has been interfaced to an embedded SoC DAQ board, exploiting a Xilinx FPGA real-time numerical elaboration, which also allows the inclusion of more sophisticated triggering mechanisms and a deeper integration with the timing systems. The feasibility of the numerical integration approach to recover the RFX magnetic field has been proved with an experimental testbed, useful to evaluate the ADC noise impact on the integration results as well. In conclusion, it has been shown that a direct digital integration appears feasible, even if it seems to require the state-of-the-art components to recover high dynamic range and wide frequency spectrum signals at the same time in a single channel. An accurate design of the input stage is mandatory; the management of the noise from the DC/DC converter is important; and the evaluation of the ADC characteristics must lead the choice of technology, matching the desired input dynamic range and the bandwidth attenuation (e.g., higher resolution ADC). The need of high quality components has been shown to be related with the 1/f low drift noise mainly coming from the ADC and the input amplifier electronics.

## Figures and Tables

**Table 1:** Cross-talk percentage, effective area and DC resistance measured for the three radial, poloidal and toroidal windings of the probe prototype.

	Radial %	Poloidal %	Toroidal %	$A_{\mathrm{eff}}$ [cm${}^{2}$]	$R_{\mathrm{DC}}\left[\Omega \right]$
Radial	/	0.28	0.07	2066	19.2
Poloidal	0.72	/	0.07	227	9.5
Toroidal	0.73	0.24	/	207	8.7

**Table 2:** RLC parameters and resonance frequency of the three prototype windings.

	Radial	Poloidal	Toroidal
Inductance [μH]	2225.16	191.11	151.94
Capacitance [pF]	163.91	302.21	308.47
Resistance [Ω]	18.88	9.65	8.05
$f_{\mathrm{res}}$ [kHz]	266.07	668.34	728.61

## References

[1] Equipe T.F.R. (1978). Tokamak Plasma Diagnostics. Nucl. Fusion.

[2] Hutchinson I.H. (2002). Principles of Plasma Diagnostics.

[3] Pustovitov V.D. (2001). Magnetic Diagnostics: General Principles and the Problem of Reconstruction of Plasma Current and Pressure Profiles in Toroidal Systems. Nucl. Fusion.

[4] Correyero S., Merino M., Elias P.Q., Jarrige J., Packan D., Ahedo E. (2019). Characterization of diamagnetism inside an ECR thruster with a diamagnetic loop. Phys. Plasmas.

[5] Amodeo M., Arpaia P., Buzio M. (2019). Integrator Drift Compensation of Magnetic Flux Transducers by Feed-Forward Correction. Sensors.

[6] Strait E.J. (2006). Magnetic diagnostic system of the DIII-D tokamak. Nucl. Fusion.

[7] Zabeo L., Ambrosino G., Cavinato M., Gribov Y., Kavin A., Lukash V., Mattei M., Pironti A., Snipes J.A., Vayakis G. (2014). Overview of magnetic control in ITER. Fusion Eng. Des..

[8] Piovesan P., Bonfiglio D., Auriemma F., Bonomo F., Carraro L., Cavazzana R., De Masi G., Fassina A., Franz P., Gobbin M. (2013). RFX-mod: A multi-configuration fusion facility for three-dimensional physics studies. Phys. Plasmas.

[9] Tumanski S. (2007). Induction coil sensors—A review. Meas. Sci. Technol..

[10] Marconato N., Bettini P., Cavazzana R., Grando L., Marchiori G., Marrelli L., Peruzzo S., Pomaro N. (2019). Design of the new electromagnetic measurement system for RFX-mod upgrade. Fusion Eng. Des..

[11] Appel L.C., Hole M.J. (2005). Calibration of the high-frequency magnetic fluctuation diagnostic in plasma devices. Rev. Sci. Instrum..

[12] Wheeler H.A. (1982). Inductance formulas for circular and square coils. Proc. IEEE.

[13] Peruzzo S., Brombin M., Palumbo M.F., Gonzalez W., Marconato N., Rizzolo A., Arshad S., Ma Y., Vayakis G., Suarez A. (2016). Progress in the Design and Testing of In-VesselMagnetic Pickup Coils for ITER. IEEE Trans. Plasma Sci..

[14] Fiorentin P., Pomaro N. (2003). Design of a new electromagnetic diagnostic for RFX. Fusion Eng. Des..

[15] Sander K.F., Reed G.A.L. (1978). Transmission and Propagation of Electromagnetic Waves.

[16] Ueda H., Watanabe T. (1975). Several Problems about Sensitivity and Frequency Response of an Induction Magnetometer.

[17] Moret J.M., Buhlmann F., Fasel D., Hofmann F., Tonetti G. (1998). Magnetic Measurements on the TCV Tokamak. Rev. Sci. Instrum..

[18] Moret J.M. (1994). Fitting of Transfer Functions to Frequency Response Measurements.

[19] Milotti E. (2002). 1/f noise: A pedagogical review. arXiv.

[20] Spuig P., Defrasne P., Martin G., Moreau M., Moreau P., Saint-Laurent F. (2003). An Analog Integrator for Thousand Second Long Pulses in Tore Supra. Fusion Eng. Des..

[21] Spuig P., Kumari P., Moreau M., Moreau P., Le-luyer A., Malard P. (2015). Enhanced integrators for WEST magnetic diagnostics. Fusion Eng. Des..

[22] Ali-Arshad S., Kock L.D. (1993). Long-Pulse Analog Integration. Rev. Sci. Instrum..

[23] Strait E.J., Broesch J.D., Snider R.T., Walker M.L. (1997). A Hybrid Digital-Analog Long Pulse Integrator. Rev. Sci. Instrum..

[24] Liu D.M., Wan B.N., Wang Y., Wu Y.C., Shen B., Ji Z.S., Luo J.R. (2009). A new low drift integrator system for the Experiment Advanced Superconductor Tokamak. Rev. Sci. Instrum..

[25] Werner A. (2006). W7-X Magnetic Diagnostics: Performance of the Digital Integrator. Rev. Sci. Instrum..

[26] Seo S., Werner A., Marquardt M. (2010). Development of a digital integrator forthe KSTAR device. Rev. Sci. Instrum..

[27] Batista A.J., Capellà L., Neto A., Hall S., Naylor G., Stephen A., Sousa J., Carvalho B., Sartori F., Campagnolo R. (2017). F4E prototype of a chopper digital integrator for the ITER magnetic. Fusion Eng. Des..

[28] Pomaro N., Basso F. (2005). Transducers and signal conditioners of the RFX new magnetic measurement system. Fusion Eng. Des..

[29] Carvalho B., Batista A., Correia M., Neto A., Fernandes H., Gonçalves B., Sousa J. (2010). Reconfigurable atca hardware for plasma control and data acquisition. Fusion Eng. Des..

[30] Manduchi G., Barbalace A., Luchetta A., Soppelsa A., Taliercio C., Zampiva E. (2012). Upgrade of the RFX-mod real time control system. Fusion Eng. Des..

[31] Stemlab–Redpitaya. https://www.redpitaya.com.

[32] Cavinato M., Manduchi G., Luchetta A., Marchiori G., Taliercio C. (2006). Distributed real time control in RFX-mod nuclear fusion experiment: Commissioning and first results. IEEE Trans. Nucl. Sci..

[33] Zuin M., Vianello N., Spolaore M., Antoni V., Bolzonella T., Cavazzana R., Martines E., Serianni G., Terranova D. (2009). Current sheets during spontaneous reconnection in a current-carrying fusion plasma. Plasma Phys. Control. Fusion.

[34] Innocente P., Zanca P., Zuin M., Bolzonella T., Zaniol B. (2014). Tearing modes transition from slow to fast rotation branch in the presence of magnetic feedback. Nucl. Fusion.

[35] Muri M.D., Cavenago M., Serianni G., Veltri P., Bigi M., Pasqualotto R., Barbisan M., Recchia M., Zaniol B., Kulevoy T. (2015). Installation and first operation of the negative ion optimization experiment. Fusion Eng. Des..

[36] Recchia M., Bigi M., Cavenago M. (2011). Conceptual design and circuit analyses for the power supplies of the NIO1 experiment. Fusion Eng. Des..

